# Promoting a sense of security in everyday life—A case study of patients and professionals moving towards co‐production in an atrial fibrillation “learning café”

**DOI:** 10.1111/hex.12955

**Published:** 2019-08-21

**Authors:** Anne‐Marie Suutari, Kristina Areskoug‐Josefsson, Sofia Kjellström, Annika M. M. Nordin, Johan Thor

**Affiliations:** ^1^ The Jönköping Academy for Improvement of Health and Welfare, School of Health and Welfare Jönköping University Jönköping Sweden; ^2^ Department of Internal Medicine and Geriatrics, the Highland Hospital (Höglandssjukhuset) Eksjö Region Jönköping County Sweden; ^3^ Department of Behavioral Sciences Oslo Metropolitan University Oslo Norway

**Keywords:** atrial fibrillation, coping behaviours, co‐production, health‐care quality improvement, patient education, Self‐Determination Theory

## Abstract

**Background:**

An improvement initiative sought to improve care for atrial fibrillation (AF) patients; many felt insecure about how to cope with AF.

**Objective:**

To reveal AF patients' and professionals' experiences of pilot‐testing a Learning Café group education programme, aimed at increasing the patients' sense of security in everyday life.

**Design:**

Using an organizational case study design, we combined quantitative data (patients' sense of security) and qualitative data (project documentation; focus group interviews with five patients and five professionals) analysed using inductive qualitative content analysis.

**Setting:**

AF patients and a multiprofessional team at a cardiac care unit in a Swedish district hospital.

**Improvement activities:**

Two registered nurses invited AF patients and partners to four 2.5‐hour Learning Café sessions. In the first session, they solicited participants' questions about life with AF. A physician, a registered nurse and a physiotherapist were invited to address these questions in the remaining sessions.

**Results:**

AF patients reported gaining a greater sense of security in everyday life and anticipating a future shift from emergency care to planned care. Professionals reported enhanced professional development, learning more about person‐centredness and gaining greater control of their own work situation. The organization gained knowledge about patient and family involvement.

**Conclusions:**

The Learning Café pilot test—exemplifying movement towards co‐production through patient‐professional collaboration—generated positive outcomes for patients (sense of security), professionals (work satisfaction; learning) and the organization (better care) in line with contemporary models for quality improvement and with Self‐Determination Theory. This approach merits further testing and evaluation in other contexts.

## 1 INTRODUCTION

### 1.1 Problem description

It was just as if they were leaving you alone on the doorstep, saying: now we are done, now you have to take care of yourself. [From a focus group interview with patients with atrial fibrillation]

With atrial fibrillation (AF) being the leading cause of emergency admissions in the local context, namely the cardiac care unit at the Highland district hospital in Region Jönköping County, Sweden, both professionals and patients experienced a need for health‐care quality improvement regarding AF management. The professionals' clinical expertise and interaction with patients and family members indicated that the support for many patients with AF was insufficient and inconsistent, causing a low sense of security in everyday life when living with AF. Due to the low sense of security in everyday life, patients felt left out, scared and unsure of how to cope with the condition, as exemplified by the quote above. Starting from the experiences of patients with AF and operating within existing resource constraints, an improvement team collaborated with patients with AF to tailor and pilot‐test a health group education model, the Learning Café. This improvement initiative aimed at increasing the patients' sense of security in everyday life.

This article reports on that improvement project and is formatted according to the Standards for QUality Improvement Reporting Excellence (SQUIRE) guidelines.^1^

### 1.2 Available knowledge

Atrial fibrillation is a common heart arrhythmia associated with an irregular heart rhythm, chest discomfort, an increased risk of stroke due to formation of thrombi inside the heart, and even of premature death. Arrhythmias in general are also associated with lowered quality of life and uncertainty, causing low patient confidence in decision making concerning treatment options and self‐care.^2^, ^3^, ^4^

To support persons living with AF, (hereafter referred to as patients with AF), clinical guidelines promote an integrated care approach with multidisciplinary teams providing easy access to appropriate care and patient support.^5^ This includes empowerment for self‐management, counselling on lifestyle changes, and risk factor management to promote coping. Coping with disease is essential for long‐term adaptation and self‐care for patients with heart disease.^6^ Problem‐focused coping strategies, that is the ability to seek information, plan and solve problems, can support decision making, decrease uncertainty due to an illness, and improve clinical outcomes and patients' quality of life.^5^, ^6^, ^7^

Inadequate patient and family support for coping with AF is common, due to insufficient disease knowledge and inadequate support in dealing with symptoms, which cause emotional distress.^8^, ^9^, ^10^, ^11^ Fear and uncertainty can be reduced when health‐care providers have time to explain AF and to help patients self‐manage their AF.^12^ Persons with a good understanding of their AF report fewer symptoms and fewer AF‐related negative emotions.^13^ Although various structured approaches to AF care have been tried worldwide, it is still unclear what design to use in different health‐care settings for integrated AF care, including patient support and education.^5^, ^14^, ^15^, ^16^, ^17^ A key design idea in such support initiatives is to enable fruitful interactions between fellow persons living with the same condition, family members and health‐care professionals. These interactions, using a non‐hierarchical structure by valuing and giving weight to each team member's views, can provide both experiential and formal disease knowledge that underpin the development of useful coping strategies.^18^, ^19^, ^20^

### 1.3 Rationale

The Learning Café is a health group education model designed on such principles to provide patients with opportunities to interact with fellow patients, family members and health‐care professionals.^21^ To improve their sense of security in everyday life, the Learning Café starts by identifying the patients' and their family members' questions and concerns. This is done to focus on what matters to them, to increase their knowledge, and to support effective coping. The Learning Café model exemplifies patient‐professional health‐care collaboration and facilitates mutual learning among patients, family members and professionals about how to live with a chronic condition.

### 1.4 Specific aim

The aim of the Learning Café initiative was to increase AF patients' sense of security in everyday life. The organizational case study reported here aimed to reveal experiences from pilot‐testing the Learning Café among participating patients and health‐care professionals.

## 2 METHODS

### 2.1 Context

The Learning Café programme was designed and pilot‐tested between September 2016 and January 2017, in the cardiac care services at the Highland district hospital in Region Jönköping County, Sweden. Serving 115 000 inhabitants in the surrounding small towns and rural areas, the cardiac services are among the hospital's largest, staffed by cardiologists, resident and intern physicians, registered and assistant nurses and a physiotherapist, an occupational therapist and administrative assistants. Each year, 300 patients with AF are admitted from the emergency department, making AF a leading cause of emergency admission at the hospital. The cardiac services operate with limited resources, which make it necessary to perform improvement initiatives within existing resource constraints.

In this particular context, there are several contextual factors that might influence quality improvement (QI) success.^22^, ^23^, ^24^ One of these factors is physician involvement (QI team leader with two additional physicians included in the improvement team). Another contextual factor promoting QI success is a high microsystem motivation to change due to an important quality gap in care identified by both patients and professionals, that is the need for improvement of AF management. The health system also has a long tradition of quality improvement work with top management leaders dedicated to constantly improve health care.^25^

### 2.2 Improvement activities

The physician leader of the cardiac care services (the first author) formed an improvement team with two additional physicians, two registered nurses specializing in cardiac care and an administrative assistant. Another nurse supported the team as Improvement Advisor. The team set out to tailor the Learning Café model to the local context. The professionals in the improvement team at the cardiac care ward invited AF patients to join the Learning Café as an additional follow‐up after a hospital admission or after an outpatient clinic visit. Patients who were invited had i) an AF‐related hospital admission or AF‐related visit at the outpatient clinic and ii) willingness to share knowledge about atrial fibrillation with fellow patients, family members and professionals. Patients unable to communicate were not asked to join the Learning Café. Ten patients, an appropriate number of patients according to the Learning Café health education model, were invited to participate. Trained to facilitate a learning café, the team's nurses organized four 2.5‐hour learning sessions with volunteering patients and partners. The Learning Café included 10 patients (7 men, 3 women) and three partners in the age range 50‐80 years. Participants had different professional and educational backgrounds. All were long‐term residents of the country. The Learning Café sessions were organized between September 2016 and January 2017 with approximately one session each month. Sessions were held at the hospital, in a dedicated room where participants were given coffee and were seated around a table with a nice tablecloth.

At the first session, the nurses solicited the participants' questions and worries about living with AF. All the patients and their partners formed smaller groups to discuss with each other. They were encouraged by the nurses to identify what matters to them and what they needed to know to feel more secure in everyday life with atrial fibrillation. Participants then wrote their questions on post‐it notes. Those notes were then shared with the professionals who were invited to discuss the questions and interact with the participants in subsequent sessions. A physician (the first author), a registered nurse on the improvement team and a physiotherapist were invited to answer the questions (Appendix S1). The administrative assistant documented the questions and answers and shared them with the participants as a record of the Learning Café sessions.

After each session and before leaving the Learning Café room, the improvement team asked the patients to rate their sense of security in everyday life with AF and their satisfaction with the Learning Café programme. The ratings were done on a scale of 0‐10, 10 representing “completely secure” and “completely satisfied”, respectively (ie self‐assessment by patients, using a non‐validated form developed by the professionals and piloted with patients). The improvement team noted that the term “sense of security in everyday life” could concern different aspects of life for different patients. The main purpose of using the ratings was to assess the patients' subjective sense of being secure despite living with AF. These ratings were reviewed by the improvement team after each session and discussed with the participants during the following sessions. Since the sense of security scale was completed anonymously at each session, no linked individual data were collected. After each session, the improvement team also reflected on the strengths of the programme and opportunities to improve it by adjusting subsequent sessions using Plan‐Do‐Study‐Act (PDSA) cycles.^26^

The introduction of the Learning Café group education programme was the team's main quality improvement intervention to improve care for patients with AF. In addition, although not further elaborated here for the sake of brevity, the team developed a checklist to guide clinicians in managing AF (ie guidance on the diagnostic workup and medical treatment) and mapped patients' care processes in co‐operation with the Learning Café participants. The checklist was only used by clinicians during patient care prior to participation of the Learning Café; therefore, it did not affect the patients' or the professionals' experience of the Learning Café initiative.

### 2.3 Study of the improvement activities, data collection and analysis

To understand the stakeholders' experiences of the Learning Café' group education programme, the authors undertook an organizational case study, using both quantitative and qualitative data.^27^ Quantitative data included patients' ratings of their sense of security in everyday life and their satisfaction with the Learning Café group education programme. These ratings were visualized graphically in chronological order. Qualitative data included documents (the project plan, notes from project meetings and field notes reflecting the improvement efforts) and transcripts from two semi‐structured focus group interviews.

All patients and professionals in the Learning Café programme were invited to the focus group interviews, scheduled a few weeks after the concluding café session. The first invitation to join the focus group interviews was made orally by the first author at the end of the last Learning Café session. The individuals interested in participating received a letter with written information about the study. Five patients and five professionals accepted the invitation to participate and formed the two interview groups, one for patients and one for professionals. Five individuals is an appropriate number of individuals to include in focus group interviews for data collection.^28^ Informed written consent was obtained from all participants. In her role as a master's student, the first author (AMS) conducted both interviews, with her master's thesis advisor (author JT) as an observer, to explore participants' experiences of the Learning Café programme. The interviews were audio‐recorded, transcribed verbatim and anonymized. The interviews were transcribed by an administrative assistant who was not part of the improvement team. Using content analysis,^29^ the main author undertook qualitative analysis according to Lundman & Hällgren Graneheim (Table 1).^30^ Since there was no pre‐existing framework expected to fit the results, she took an inductive approach to the content analysis.^31^ The draft analysis was reviewed with the advisor (JT) and finalized by reaching consensus as a form of investigator triangulation. The validity of the case study was strengthened by using data and informant triangulation, combining quantitative and qualitative data, and by illustrating interview themes with quotes from both patients and professionals to reveal different perspectives.^27^

**Table 1:** An example of a meaning unit, code, subcategory, category and theme as postulated by Lundman & Hällgren Graneheim^30^

Meaning unit	Code	Subcategory	Category	Theme
*I am, at base, a thinking and worrying person, a bit anxious you know. [‐‐] So, when I was diagnosed with atrial fibrillation, I felt quite clearly that there is too little information about what it means, what the risks are, how am I supposed to handle it, is it something that comes and goes, or will it remain forever and so on, so there was a lot of information that I missed. So, this has really been a wonderful thing, I think*	Need for information	Meet the information needs	Facilitation of coping strategies	The persons living with atrial fibrillation: Quality of life

### 2.4 Ethical considerations

Written informed consent was obtained from all participants prior to data collection. The study was vetted by the Regional Research Ethics Review Board in Linköping, Sweden (Dnr 2016/493‐31).

## 3 RESULTS

### 3.1 Patients' ratings

**Figure 1:**
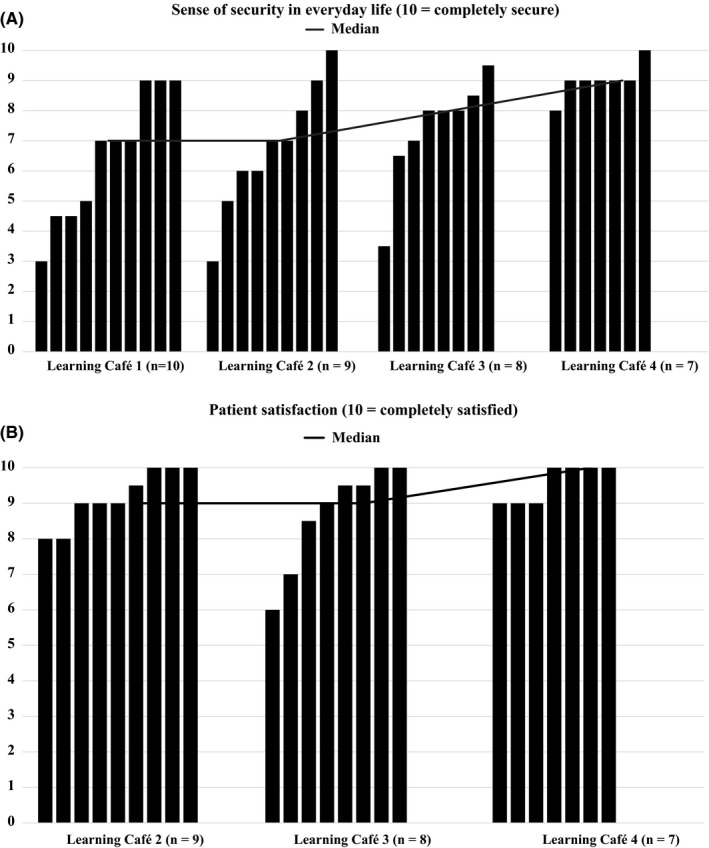
(A) Sense of security in everyday life. (B) Patient satisfaction. Each bar represents a patient in the Learning Café group education programme. The black line represents the median ratings. The rating for the sense of security at the first Learning Café session was considered to be the baseline rating. Patient satisfaction was only rated after Learning Café sessions 2‐4, with invited health‐care professionals, to evaluate the patients' experience of the format used then to discuss the participants' questions

The patients' sense of security in everyday life increased (median rating increasing from 7 to 9) with successive sessions (Figure 1). The patients' satisfaction with the Learning Café sessions was high throughout the programme (median rating increasing from 9 to 10). Some quantitative data concerning the patients' sense of security and satisfaction were missing since some patients could not participate in all four sessions. No new participants joined after the first session.

### 3.2 Patients' and professionals' experiences

The participants' questions solicited during the first Learning Café session revealed what mattered to these patients with AF and their family members. The questions concerned disease mechanisms, triggers, treatment, self‐care, physical activity and when and how to access health care (Appendix S1). Based on the amount of questions reflecting the patients' concerns and the intense discussions among patients, family members and professionals, the last three categories, self‐care, physical activity and when and how to access health care, were clearly especially important for patients' sense of security in everyday life.

Three themes emerged from the content analysis of the focus group interviews, reflecting different perspectives on the Learning Café programme (Table 2). These themes were as follows: the patients' experience of improved quality of life, the health‐care professionals' enhanced working experience, and the organization's change in health‐care delivery.

**Table 2:** Themes and categories

Themes	Categories
The patients with atrial fibrillation: Quality of life	Strengthening coping strategies Positive health experience
The health‐care professionals: Working environment	Work satisfaction Professional development
The organization: Delivery of health care	Patient/family involvement Possible change in health‐care utilization

#### 3.2.1 The patients with atrial fibrillation: quality of life

The patients with AF reported a lack of knowledge regarding their medical condition and how to handle it, leaving them feeling insecure in everyday life. The professionals confirmed that this was a quality gap in routine care due to time shortages and a lack of forums for providing sufficient support. Both patients and professionals said that the Learning Café created conditions for sharing of knowledge and patient support, thus facilitating coping and increasing patients' sense of security in everyday life. The sharing of knowledge also promoted the professionals' learning about the patients' perspectives on living with chronic AF, thus leading to mutual learning and growth in both groups:

I am, at base, a thinking and worrying person, a bit anxious you know. [‐‐] So, when I was diagnosed with atrial fibrillation, I felt quite clearly that there is too little information about what it means, what the risks are, how I am supposed to handle it, is it something that comes and goes, or will it remain forever and so on, so there was a lot of information that I missed. So, this has really been a wonderful thing, I think. [Focus group interview with AF patients, FGP]

As a physician I always experience that you don't have enough time at the outpatient clinic to address all the questions and the things that the patient needs to know [‐‐] So, they [the patients] do not get enough information, not at the cardiac ward either, so it feels really great that there is this opportunity for them to get this information that reassures them. [Focus group interview with staff members, FGS]

Patients and professionals noted the opportunity to share knowledge and concerns with family members and fellow patients as an improvement compared to traditional care, mostly involving individual visits and less interaction. Respondents indicated that the “Learning Café” promoted meaningful relationships and contributed to a positive health experience for AF patients:

It really is a fantastic thing that they [family members] can join and share the information with their partners [‐‐] because sometimes you [as a patient] can't recount all of what has been said and then, the family members usually ask a lot of questions and then if you don't have answers to them… [FGP]

…there is this thing with sharing your concerns, you share your issues with so many others and you realize that everybody has the same questions, everybody has the same concerns, everybody has the same need for answers. We are not alone. [FGP]

…It is different from having individual visits because then it is only just that person and it [the advice given] might be up to that health care professional sitting with the patient, but here, the patients gave each other some advice… [FGS]

#### 3.2.2 The health‐care professionals: working environment

The expectation was that improving care through the Learning Café' initiative would enhance work satisfaction and facilitate professional development. The quotes below illustrate the team members' improved knowledge about person‐centredness and increased awareness regarding the importance of meaningful interactions between professionals and patients. The quotes also indicate team members' satisfaction with gaining control of their own work situation:

We've had the chance to see them not as patients, but as persons. [FGS]

It has felt like an energy boost also for us, it is like you're reminded of why you chose to work with this in the first place [‐‐] but you get so incredibly much back from the patients and you really have the time to see them as individuals. So that's like a reward too. [FGS]

You don't just stand still but you do something about an existing problem, you work with others who want things to get better too instead of just complaining about what is working poorly. [FGS]

#### 3.2.3 The organization: delivery of health care

Prior to launching the Learning Café, the professionals worried that it might fail to convey important aspects of AF if they did not control the content of the group sessions. During the improvement effort, they gained new insights concerning patient and family member involvement—to start with what mattered to them shifted the focus from what information health‐care professionals wanted to pass on to patients, to what patients sought and needed. This did not mean that important information was missed out:

It is interesting because it is almost like you start with the patient's problem [‐‐] Because I think that often when you see the patient, there is a big focus on what the healthcare system wants to get out of the visit [‐‐]and it is not always the patient's real problem that is in focus. [FGS]

We were a bit worried that they [the patients and family members] would not ask for certain things since we wanted to get across a particular type of information. But I think we actually did here. [FGS]

Both patients and professionals reported that participating in the “Learning Café” created a sense of security in everyday life that could change patients' patterns of future health‐care utilization in a welcome way, shifting from unplanned emergency care to planned care:

When you experience the reassurance from getting answers to a lot of questions, you don´t need to make emergency calls so often. [FGP]

I called 112 [the emergency number] and that is what I have done twice before when I have felt it [atrial fibrillation] really hard. But now I have waited the last couple of times [and] it [the spell of atrial fibrillation] has ended spontaneously… [FGP]

And I also think that when the patients' sense of security increases, they might choose to wait a little bit and follow the recommendations instead of getting in touch with the emergency department immediately. [FGS]

**Figure 2:**
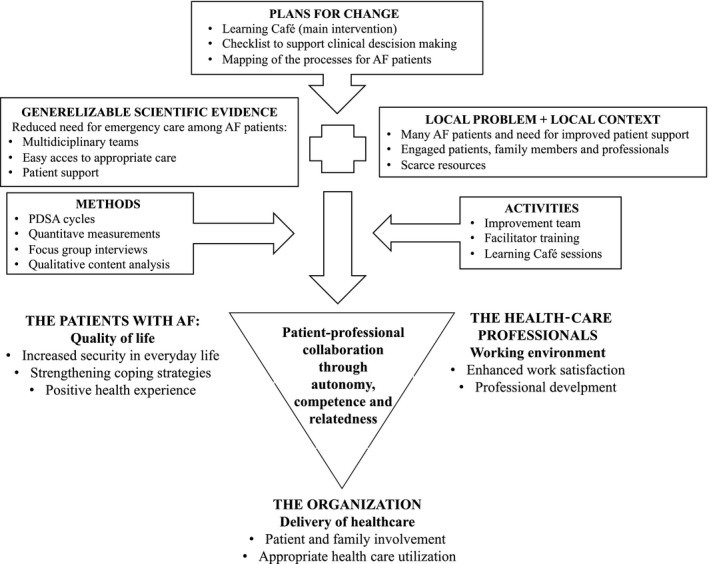
The Learning Café pilot test—exemplifying early steps on the way towards co‐producing health and health care through stakeholder autonomy, competence and relatedness – generating positive outcomes for patients, professionals and the organization (modified from Batalden & Davidoff^32^)

Applying existing quality improvement models^32^, ^33^ on this case study, the next section synthesizes the pilot test experiences into a conceptual model. The model illustrates and summarizes the improvement efforts and the findings of the Learning Café case study (Figure 2).

## 4 DISCUSSION

The context‐ and culture‐specific results from this study indicate that the Learning Café pilot test—exemplifying collaboration between patients and professionals in health care—generated positive outcomes for patients, professionals and the organization. The patients with AF gained a greater sense of security in everyday life indicating strengthened coping strategies. The professionals reported enhanced professional development, learning more about person‐centredness and gaining greater control of their own work situation when being able to perform improvement work within existing resource constraints. The organization gained knowledge about how to involve patients and family members in health care. The anticipated reduced need to seek emergency care reported in the study indicates a promising future shift from emergency care to planned care.

Reflecting on the findings after the conclusion of the data analysis, we found that innate psychological intrinsic motivators included in Self‐Determination Theory (SDT)^34^, ^35^—autonomy, competence and relatedness—can link the execution of the Learning Café programme to its results. Autonomy represents peoples' need to feel that they have choices and that their behaviours are self‐endorsed.^35^ Competence refers to peoples' need to develop mastery and to operate effectively within their own lives.^35^ Relatedness concerns peoples' need to care about, and be cared for by, others.^35^ SDT posits that people, in this case patients with AF and professionals, will naturally engage in interesting, challenging and enjoyable activities, which help satisfy these innate psychological needs.^36^ Previous research implies that the drive to satisfy these needs greatly influences if and how patients participate in service development in health care.^37^ SDT could, therefore, help explain what motivates stakeholders to collaborate in health care and, thereby, to promote self‐management, well‐being and work satisfaction.^34^, ^35^ Applying the components of SDT retrospectively to the results thus helps us make sense of our research findings.^38^

### 4.1 Learning Café strengthening coping strategies

One explanation for the increase in patients' overall sense of security in everyday life with successive Learning Café sessions (Figure 1) could be that the programme provided an opportunity to share knowledge and personal experience, serving to increase their competence regarding the disease and its treatment. Furthermore, the programme created conditions for interacting with—relating to—fellow patients, family members and knowledgeable health‐care staff. Competence and relatedness can facilitate coping and promote patient autonomy.^12^, ^13^, ^18^, ^19^, ^34^, ^39^, ^40^, ^41^, ^42^, ^43^, ^44^ Some participating patients reported actual or anticipated changes in their patterns of health‐care utilization, expecting that they would refrain from going to the emergency room so quickly, due to the Learning Café experience. This indicates that facilitating coping strategies might reduce patients' demand for emergency care. This aligns with research suggesting that patient support, one of the components of integrated AF care, can reduce the demand for emergency care among patients with AF.^5^, ^14^, ^15^, ^16^

### 4.2 Learning Café enhancing working experience

The team members, working in a context characterized by resource constraints, reported enhanced work satisfaction and professional development when working with the education programme. They appreciated gaining control of their own work situation because, in SDT terms, it promoted their autonomy. Their statements about seeing patients as persons indicate increased competence regarding person‐centredness. Furthermore, their enhanced work satisfaction from positive patient feedback indicates the importance of interaction and relatedness. Previous research supports the idea that organizations benefit from creating working environments that promote intrinsic motivators. Promotion of intrinsic motivators includes focusing on the meaningfulness of work, on personal mastery, on providing positive feedback, on opportunities to learn new things, and contributing to quality improvement.^45^, ^46^, ^47^, ^48^, ^49^, ^50^

### 4.3 Learning Café—a person‐centred approach to co‐production of health care

Loeffler et al^51^ define co‐production of health‐care services as collaboration between patients, family members and health‐care professionals at many stages of the health‐care process: (a) co‐planning services, including co‐prioritization of health care; (b) co‐design of health care; (c) co‐delivery of health care including co‐managing and co‐performing health care and (d) co‐assessment including co‐monitoring and co‐evaluation of health care. Osborne et al^52^ state that the role of learning in co‐production deserves more attention, that is learning about how to co‐produce effectively and how lessons from co‐production could be used for service improvement. Building on these statements, co‐production of health care is understood in this paper as when patients, family members and professionals collaborate along the health‐care process and learn together to further improve health‐care services.

The professionals participating in the study context lacked previous experience of how to involve patients in co‐producing improvement efforts. Therefore, the initial planning phase involved only professionals, who thereafter invited patients to join the Learning Café process, thus collaborating on the remaining steps of the improvement effort. Through their questions, patients determined the content of the sessions, exemplifying co‐design and co‐delivery of the Learning Café. The patients interacted with other participants and health‐care professionals, indicating co‐delivery of the service. By rating the sense of security in their everyday life, their satisfaction with the Learning Café sessions and by discussing these ratings together with the professionals, the patients were involved in co‐evaluation. By asking new questions and by sharing knowledge with professionals and fellow participants, there was co‐learning among patients, partners and professionals regarding what matters when living with chronic AF and how to handle the condition in everyday life.

Thus, although not including every possible aspect of co‐production of health care, the Learning Café—involving patients and family members, starting with what matters to them, using a person‐centred approach—exemplifies movement towards co‐production of health‐care services in the present context through patient‐professional collaboration.^51^, ^52^, ^53^, ^54^ We suggest that this initiative promoted the patients' and professionals' autonomy, competence and relatedness. Previous research implies that the drive to satisfy these fundamental human needs greatly influences if and how patients participate in service development in health care.^37^ Slay & Penny^55^ draw further attention to the relationship between co‐production of health care and these intrinsic motivators. When professionals can become care facilitators rather than care givers, it enhances both patients' and professionals' autonomy. Seeing people as partners and building on stakeholders' pre‐existing capabilities can enhance the patients' and the professionals' competence. Promoting relationships between professionals and patients and engaging personal networks, for example family members, enhances the patients' and professionals' relatedness.

Batalden & Davidoff^32^ suggest that applying generalizable scientific evidence—in this case patient support initiatives as a part of an integrated care approach for patients with AF—requires knowledge about what works and how to make it work in a particular context. Recently, Batalden also suggested that knowledge about the patient's aim, that is the reason for seeking help, is equally fundamental to improving health care.^33^ Both papers suggest that health‐care improvement efforts integrate better patient outcomes (health), better organization performance (care) and better professional development (learning; joy in work).^32^ Combining these propositions with SDT and insights from this Learning Café case study, Figure 2 offers a model for improvement efforts with empirical examples from the case.

### 4.4 Methodological considerations

This is a single case study of a pilot test in one particular setting. That obviously limits the generalizability of its findings on the usefulness of the Learning Café model. Further testing in this and other contexts will add valuable knowledge on how best to design health care through the model.

We sought to strengthen the case study's validity through data and informant triangulation, drawing on complementary data collection and analysis methods.^27^, ^55^ The interviewer and first author worked in the local context and led the improvement team. While being immersed in the research field enabled her to interpret data in light of the local context, being an insider may also have shielded her from some perspectives. Data analyses were reviewed with a senior improvement researcher (author JT) adding an outsider perspective as a form of investigator triangulation.

All the professionals participated in the focus group interview, and five of the 10 patients joined. The professionals may have felt obligated to participate. They appeared, however, to join willingly, reflecting the spirit of the team. Among the patients, there may have been selection bias if only patients with positive experiences decided to participate. We have no indications of this being the case, since the satisfaction ratings were overwhelmingly positive from all participants. There is a risk of bias due to the authors' preunderstanding and close relationships with the participants, particularly the risk of “social desirability”.^56^ To minimize this bias, all patients were encouraged to leave anonymous written comments on the education programme instead of, or to complement, ratings and interview participation. Only one patient did so and proposed changes to improve future Learning Café sessions. The proposed suggestions were about starting a group only for women and the necessity for Learning Café leaders to encourage everybody to talk equally much. Mindful of these limitations, we invite readers to build on knowledge from this case study about health‐care collaboration between patients, family members and professionals.

Another limitation, reflecting the pragmatic nature of this initiative, relates to how patients self‐assessed their sense of security and satisfaction, using the locally developed form. Although not validated, the form provided a useful measurement tool to guide these improvement efforts. Some patients were not able to participate in all four sessions, which was reflected in the quantitative data. We cannot know if these measurements would have differed from those recorded but have no indications to suggest so. Having a patient research partner involved in co‐producing the analysis would have been potentially beneficial in providing an additional perspective to the results.

We were not able to collect follow‐up data on the programme from participants after its conclusion; therefore, we cannot report on the sustainability of its favourable effects. Future research could include such longer‐term follow‐up to assess both patients' sense of security and their actual demand for emergency services over time.

## 5 CONCLUSIONS

The pilot test of the Learning Café group education programme exemplifies movement towards co‐production through patient‐professional collaboration with enhanced stakeholder autonomy, competence and relatedness. The pilot test generated positive outcomes for patients (sense of security), professionals (work satisfaction; learning) and the organization (better care) in a Swedish district hospital setting. The improvement initiative was conducted within existing resource constraints. Since the completion of our pilot test, top management in the health system has promoted Learning Cafés with additional patient groups and in different contexts, that is not only within specialized care but also within primary care contexts. Further testing and research are warranted to understand the sustainability of the results and how the Learning Café group education programme could be enhanced and applied beneficially in other contexts and with other groups of patients. Further testing and research are also warranted to explore whether this improvement initiative can reduce the patients' need for emergency care in favour of planned care.

## CONFLICT OF INTEREST

The authors declare that they have no conflicts of interest in publishing this work.

## PATIENT CONSENT

Informed consent was obtained from the participants prior to data collection.

## ETHICAL APPROVAL

The study was vetted by the Regional Research Ethics Review Board in Linköping, Sweden (Dnr 2016/493‐31).

## Data Availability

Research data at the School of Health and Welfare, Jönköping University, Sweden, are regulated under the Freedom of the Press Act (1949:105) and the Public Access to Information and Security Act (2009:4) as Public Records/Official Documents. Research data that is registered and Archived at the School of Health and Welfare can be requested anonymously by anyone for a fixed fee according to the Fee Regulation (1992:191). If the research data contain sensitive information, like personal data and/or trade secrets, that information is protected by Confidentiality and Secrecy by the Public Access to Information and Security Act and requests of such information will be denied with the possibility to appeal the denial in the Court of Appeals. Secret or Confidential research data can be accessed by other researchers if they receive permission from the Regional Ethics Review Board. The lawful basis for transmission of Secret and/or Confidential information is then based on the Law of Ethics Review for Research on Humans (2003:460).
