# Supplementary material for: Promoting a sense of security in everyday life—A case study of patients and professionals moving towards co‐production in an atrial fibrillation “learning café”
Source: Health Expect. 2019 Aug 21;22(6):1240–50. doi: 10.1111/hex.12955 (PMC6882262; doi:10.1111/hex.12955)
Supplement: Supplementary file 1 [file HEX-22-1240-s001.docx]

# **APPENDIX S1: LEARNING CAFÉ QUESTIONS FROM PARTICIPANTS**

# For how long has atrial fibrillation (AF) been known? How was the disease found from the beginning?

- Is AF hereditary?
- Are there ways to prevent AF?
- How common is the disease (AF)?
- Has the number of patients suffering from AF increased? Is it more common now? Is there any link with the stress in society?
- What is the mean age for women when diagnosed with AF?
- Are there any ongoing research projects regarding AF?
- Are there any factors/circumstances that triggers AF?
- Is there any link between crises and stress and AF?
- Facts about AF?
- Possibilities to impact AF?
- Cardioversion – how and when?
- Does the presentation of AF differ between individuals?
- What can I do by myself to terminate an attack?
- What can make AF worse?
- What should you avoid?
- Is there any final help to get rid of AF?
- Should you massage your neck when you have AF?
- Why should I wait before getting in contact with healthcare when I suffer from an AF attack?
- For how long should you wait before you call (the healthcare professionals) when you have an AF attack?
- Who should I contact when I need help, e.g. to get a cardioversion?
- Who should I contact when I need help with anxiety and to get answers to my questions?
- Telephone contact with a doctor, how?
- Why do I wake up at night with a pounding pulse (the pulse frequency is normal)? I am sweating at the same time.
- If I lay on my left side, I notice that the heart beats hard in a different way even if the rhythm is stable and I do not have AF at the moment, why?
- If I lay on my right side, I can trigger an AF attack, why? Is it because there is more strain on the heart/body when I lay on my right side?
- Are there different kinds of AF since we all have so different symptoms?
- Has my dizziness to do with the AF?
- Is AF a serious disease?
- Why does the normal rhythm of the heart change to AF?
- Why does it feel like the heart “turns a volt”?
- Is there an association between the number of heart beats and how long you live?
- Is there an association between blood pressure and AF?
- Could influenza be a trigger for AF?
- How many times can you get a cardioversion?
- Is it dangerous to get a cardioversion many times?
- What is required to be considered for cardiac ablation?
- Can you have cardiac ablation several times?
- Pacemaker – can that be a solution for AF?
- What are my right for sick leave when I suffer from AF?
- Are there any restrictions for travelling abroad?
- Are there any restrictions for travelling by plane?
- If you are going away for 14 days, what should you think about? What happens if you get an AF attack while being away?
- Warfarin or another drug, which drug is the best?
- How to handle anxiety regarding bleedings?
- What about follow-ups?
- Antidote for Eliquis^®^?
- Omeprazole to prevent bleedings when taking anticoagulants?
- How does Cordarone^®^ work?
- Why do we have different medicines and why do different medicines help unevenly?
- Is there any association between night mares and new AF medicines?
- Low blood pressure when you take betablockers causing dizziness and black for the eyes?
- Can physical exercise trigger AF?
- Can stress trigger AF?
- Can electricity/radio waves/cellular phones affect the electrical activity in the heart thereby causing AF?
- What is inappropriate to do at the gym?
- Is there any physical activity that you should avoid when suffering from AF?
- Is it dangerous to have a physically strenuous work when you suffer from AF?
- Is it easier to get AF if you are stressed?
- Why do I sweat more nowadays?
- Does sleep affect AF?
- Can you exercise as much as you want when you suffer from AF?
- Is there any association between AF and doing sports on a high lever earlier in life?
- How much alcohol do you dare to drink?
- What about food?
